# Sexual dysfunction, depressive symptoms, and perceived impact of HBV infectivity among noncirrhotic patients with chronic hepatitis B: a cross-sectional study

**DOI:** 10.3389/fpubh.2026.1906589

**Published:** 2026-09-29

**Authors:** Chengrun Song, Jing Zhou, Jiangnan Peng, Yujing Li, Sahar Muzammal, Enqiang Chen

**Affiliations:** 1 Center of Infectious Diseases, West China Hospital of Sichuan University, Chengdu, Sichuan, China; 2 Department of Cardiology, West China Hospital, Sichuan University, Chengdu, Sichuan, China

**Keywords:** chronic hepatitis B, depressive symptoms, HBV infectivity, pegylated interferon α-2b, quality of life, sexual dysfunction

## Abstract

**Background:**

Chronic hepatitis B (CHB) remains a major global public health burden, yet sexual health is rarely addressed in routine care. This study aimed to assess the prevalence of sexual dysfunction (SD) and examine demographic, clinical, and psychological factors associated with sexual function among noncirrhotic patients with CHB. We also explored the association between sexual function and the perceived negative impact of HBV infectivity on sexual life.

**Methods:**

This cross-sectional study was conducted from December 2023 to July 2024, enrolling 657 patients with CHB and 121 non-HBV controls. Patients with CHB were classified by treatment status as receiving pegylated interferon α-2b (*n* = 391), receiving nucleos(t)ide analogues (*n* = 203), or not receiving antiviral therapy (*n* = 63). Sexual function was assessed using the IIEF-5, EHS, and PEDT in men and the FSFI in women. Psychological status was assessed with the PHQ-9 and GAD-7, and the perceived negative impact of HBV infectivity on sexual life was assessed using a single exploratory yes/no item. Hierarchical multiple linear regression was used to identify factors associated with sexual function scores.

**Results:**

The prevalence of SD was significantly higher in all CHB subgroups than in Non-HBV group, with the highest prevalence observed in the IFN-CHB group, both in men (70.9%) and women (70.8%). In multivariable models, older age, IFN-CHB status, and higher PHQ-9 scores were associated with lower sexual function scores in both sexes. Of the variables examined, IFN-CHB status showed the strongest negative association in men (*β* = −0.267, *p* < 0.001), whereas PHQ-9 score showed the strongest negative association in women (*β* = −0.312, *p* = 0.006). A perceived negative impact of HBV infectivity on sexual life was reported by 21.6% of men and 24.7% of women with CHB. Men reporting this perception had lower IIEF-5 scores (*p* < 0.001), and women reporting it had lower FSFI scores (*p* = 0.020).

**Conclusion:**

Sexual dysfunction was common among sexually active patients with CHB, and poorer sexual function was associated with IFN-CHB status, depressive symptoms, and the perceived negative impact of HBV infectivity on sexual life. Sexual health assessment and mental health support may therefore have value in comprehensive CHB care.

## 1 Introduction

Chronic hepatitis B virus (HBV) infection remains a major global public health challenge, affecting approximately 240 million people worldwide (1). With the wider use of effective antiviral therapy, durable virological suppression and prevention of liver complications have become increasingly achievable for chronic hepatitis B (CHB) patients. However, quality of life in this population remains insufficiently recognized and addressed. Sexual dysfunction (SD) is particularly important in this context because it may impair quality of life, intimate relationships, and treatment experience (2–4). The development of SD in CHB is likely multifactorial, involving endocrine changes, treatment factors, and psychosocial stressors. Alterations in sex hormone metabolism and hypothalamic pituitary gonadal axis (HPG) regulation may contribute to impaired sexual function (5). Beyond these biological factors, living with CHB may be accompanied by stigma, fatigue, reduced social functioning, and impaired quality of life. Concerns about infectivity, depressive symptoms, reduced sexual confidence, and avoidance of sexual activity may further compromise sexual health (2, 6).

Sexual health, as defined by the World Health Organization, encompasses physical, emotional, mental, and social well-being in relation to sexuality, rather than merely the absence of disease or dysfunction. Despite this broader public health perspective, existing evidence on sexual function in CHB remains limited and unevenly distributed. Previous studies mainly focused on male erectile dysfunction (ED), with reported prevalence estimates varying substantially according to disease stage, age distribution, and diagnostic criteria (7–9). In contrast, female sexual function in CHB has been less systematically investigated, limiting a comprehensive understanding of the sexual health burden in this population (4, 10). In addition, although antiviral therapy is central to CHB management, the potential association between treatment modality and sexual function has not been well characterized. Nucleos (t)ide analogues (NAs) are generally well tolerated, although evidence regarding their association with sexual function remains insufficient for firm conclusions (11, 12). Pegylated interferon-α (Peg-IFN-α) may be accompanied by systemic and neuropsychiatric adverse effects, such as fatigue, myalgia, mood symptoms, and reduced well-being, which could plausibly affect sexual health (13, 14). Clinically, patients frequently report sexual disturbances coinciding with antiviral treatment initiation, yet it is unclear whether these issues stem from the pharmacological properties of the drugs, the physiological stress of the underlying disease, or pre-existing psychological vulnerabilities.

Comparative data across treatment-defined CHB populations are therefore needed to clarify the burden and correlates of SD in this setting. This observational cross-sectional study aimed to characterize the prevalence of SD among men and women with CHB stratifying by treatment status (Peg-IFN-α, NAs, and treatment-naïve). Using validated instruments for sexual function and depressive symptoms, together with clinical parameters, we further explored factors associated with SD. We also examined the association between sexual function and the perceived negative impact of HBV infectivity on sexual life. These findings may help inform more patient centered management by highlighting the need to consider sexual health, depressive symptoms, and perceived infectivity-related concerns in routine care.

## 2 Methods

### 2.1 Study design and setting

A cross-sectional observational study was conducted between December 2023 and July 2024 across eight hepatology outpatient clinics in China. At each clinic, patients with CHB were consecutively screened against prespecified eligibility criteria. All eligible patients were invited to participate, and those who agreed completed the questionnaire. The numbers recruited and included in the primary analysis at each site are presented in Supplementary Table S1.

### 2.2 Participants and sampling

The study populations comprised adult patients aged 18 to 60 years with a confirmed diagnosis of CHB, categorized by antiviral treatment status into three groups: (1) pegylated interferon-α-2b treated (IFN-CHB, *n* = 391), (2) nucleos(t)ide analogues treated (NAs-CHB, *n* = 203), (3) treatment-naïve patients (TN-CHB, *n* = 63). Non-HBV participants, enrolled as a reference group (*n* = 121), were recruited from health examination attendees, hospital staff, and community volunteers. They were required to meet the same age range as the CHB participants and have documented HBsAg negativity in previous health examination reports or medical records.

Participants reporting sexual inactivity, defined as no sexual activity during the previous three months, were excluded from the primary IIEF-5 and FSFI analyses because valid interpretation of these scores requires recent sexual activity. Additional exclusion criteria were invalid questionnaire and major comorbidities that could substantially affect sexual function, including diabetes mellitus, uncontrolled hypertension, coronary artery disease, malignancy, and severe psychiatric disorders.

The sample size was calculated to estimate the prevalence of sexual dysfunction among patients with CHB. Based on pilot data from our center, the expected prevalence was approximately 60%. With a 95% confidence level, an absolute precision of 5%, and an assumed 10% rate of incomplete or invalid questionnaires, at least 410 patients with CHB were required. No fixed ratio between CHB patients and non-HBV participants was prespecified.

### 2.3 Ethics statement and data collection

Ethical approval for this study was obtained from the Biomedical Ethics Committee of West China Hospital, Sichuan University, and the study protocol complied with the principles of the Declaration of Helsinki. The requirement for written informed consent was waived by the ethics committee because the study used anonymous online questionnaires and did not collect personally identifiable information. Before completing the questionnaire, participants were informed of the study purpose, procedures, voluntary nature of participation, and their right to withdraw at any time. Participants proceeded to the questionnaire only after clicking a confirmation button indicating their voluntary participation. Data were collected using structured online questionnaires administered through Wenjuanxing. Regular exercise was defined as at least 75 min of physical exercise per week. Smoking was defined as at least one cigarette per day in the last 6 months and drinking was defined as at least once a week in the last 6 months. All questionnaire items were mandatory. Only one submission was permitted per IP address to minimize duplicate submissions. Submitted questionnaires containing responses with logical inconsistencies were classified as invalid and excluded.

### 2.4 Assessment tools

Sexual function was quantitatively assessed using validated, self-administered questionnaires. For male participants, erectile function was evaluated using the Erection Hardness Score (EHS) and the 5-item International Index of Erectile Function (IIEF-5). An EHS < III and an IIEF-5 score ≤ 21 were defined as indicative of erectile dysfunction (ED) (15, 16). Premature ejaculation (PE) was assessed using the Premature Ejaculation Diagnostic Tool (PEDT), with a score ≥ 11 serving as the diagnostic cutoff (17). For female participants, the 19-item Female Sexual Function Index (FSFI) was administered, with a total score ≤ 26.55 used to define female sexual dysfunction (FSD) (18, 19). Psychological status was evaluated using the Patient Health Questionnaire-9 (PHQ-9) for depressive symptoms and the Generalized Anxiety Disorder-7 (GAD-7) scale for anxiety symptoms (20, 21). Perceived negative impact of HBV infectivity was assessed among participants with CHB using a single self-reported yes/no item: “Do you worry about or avoid sexual activity because hepatitis B is an infectious disease?” Participants who answered “yes” were classified as having perceived negative impact of HBV infectivity on sexual life.

### 2.5 Statistical analysis

Data analysis was performed using SPSS version 26.0 (IBM Corp., Armonk, NY, USA) and R version 4.5.2 (R Foundation for Statistical Computing, Vienna, Austria). Normality of continuous variables (age, BMI, duration of HBV diagnosis, PHQ-9, GAD-7, IIEF-5, FSFI total, and FSFI domain scores) was assessed using the Shapiro–Wilk test and visual inspection of histograms and Q-Q plots. Continuous variables were summarized as mean ± standard deviation or median (interquartile range). Categorical variables were summarized as frequencies and percentages, and intergroup comparisons were conducted using the Chi-square test or Fisher’s exact test, as appropriate. For comparisons among the four groups, one-way ANOVA or the Kruskal-Wallis H test was employed, followed by post-hoc pairwise comparisons with Bonferroni correction if significant differences were detected. Hierarchical multiple linear regression models were constructed with IIEF-5 scores in men and FSFI total scores in women as dependent variables. Candidate variables included those with *p* < 0.10 in univariable analyses and demographic or clinically relevant variables considered important for sexual function. Variables were entered in blocks: Model 1 included demographic and lifestyle variables, Model 2 additionally included study group, and Model 3 further included PHQ-9 and GAD-7 scores. For multivariable linear regression models, assumptions were evaluated using normal P–P plots of standardized residuals and scatterplots of standardized residuals against standardized predicted values, and multicollinearity was assessed using variance inflation factors (VIFs). Linear mixed-effects models with site as a random intercept were fitted to assess potential site effects. Regression coefficients with 95% confidence intervals (CI), R^2^, and ΔR^2^ were reported. Multivariable logistic regression analyses were conducted to examine the association between study group and sexual inactivity, adjusting for age, marital status, education level, PHQ-9 score, and GAD-7 score, and adjusted odds ratios with 95% CIs were reported. All statistical tests were two-tailed, and *p* < 0.05 was considered statistically significant.

## 3 Results

### 3.1 Participant characteristics

During screening, 134 individuals reporting no sexual activity in the previous three months were excluded. Their distribution by sex and group is shown in Supplementary Table S2. Finally, a total of 778 participants were included in the analysis, comprising 657 patients with chronic hepatitis B and 121 Non-HBV controls. Among patients with CHB, 391 were receiving pegylated interferon α-2b therapy (IFN-CHB), 203 were receiving nucleos(t)ide analogues therapy (NAs-CHB), and 63 were treatment naïve (TN-CHB). Baseline characteristics are summarized in Table 1. The four groups were comparable in body mass index, marital status, and smoking status. However, age differed across groups, with the highest mean age observed in the NAs-CHB group (43.34 ± 9.08 years) and the lowest in the TN-CHB group (37.97 ± 7.96 years). The Non-HBV control group had a higher proportion of participants with college education or above. Alcohol use also differed significantly among groups, with lower reported alcohol use among patients with CHB than among Non-HBV controls. In sensitivity analyses, sexual inactivity varied across the four groups and was most frequent in the IFN-CHB group (12.7%, 44/346), compared with the NAs-CHB group (2.6%, 4/151), the TN-CHB group (0%, 0/42), and the Non-HBV group (3.5%, 3/86) (*p* < 0.001; Supplementary Table S2).

**Table 1:** Characteristics of the study population.

Characteristics	IFN-CHB (*n* = 391)	NAs-CHB (*n* = 203)	TN-CHB (*n* = 63)	Non-HBV (*n* = 121)	*P* value
Age, year	40.77±7.74	43.34±9.08	37.97±7.96	39.53±6.91	<0.001
Sex, *n* (%)					
Female	89 (22.8%)	56 (27.6%)	21 (33.3%)	38 (31.4%)	0.114
Male	302 (77.2%)	147 (72.4%)	42 (66.7%)	83 (68.6%)	
BMI, kg/m^2^	23.25±2.98	23.17±3.74	22.60±2.51	23.25±2.80	0.497
Education level, n (%)					<0.001
Secondary school	66 (16.9%)	38 (18.7%)	9 (14.3%)	2 (1.7%)	
High school	141 (36.1%)	75 (36.9%)	17 (27.0%)	10 (8.3%)	
University or above	184 (47.1%)	90 (44.3%)	37 (58.7%)	109 (90.1%)	
Marital status, *n* (%)					0.243
Unmarried	24 (6.1%)	11 (5.4%)	9 (14.3%)	8 (6.6%)	
Married	354 (90.5%)	189 (93.1%)	53 (84.1%)	111 (91.7%)	
Divorced	13 (3.3%)	3 (1.5%)	1 (1.6%)	2 (1.7%)	
Smoking status, *n* (%)	57 (14.6%)	23 (11.3%)	9 (14.3%)	15 (12.4%)	0.713
Alcohol use, *n* (%)	6 (1.5%)	5 (2.5%)	0 (0.0%)	13 (10.7%)	<0.001
Regular exercise, *n* (%)	83 (21.2%)	59 (29.1%)	16 (25.4%)	39 (32.2%)	0.046
Duration of HBV diagnosis, years	20 (10, 25)	20.00 (15, 30)	15 (4, 23)	-	0.001
History of multi-drug therapy	51 (13.0%)	28 (13.8%)	-	-	0.800
PHQ-9 score	6 (3, 10)	5 (2, 9)	7.00 (3, 9)	5 (1, 7)	0.005
GAD-7 score	3 (0, 7)	3 (0, 7)	4.00 (1, 7)	3 (0, 6)	0.160

IFN-CHB, chronic hepatitis B patients receiving pegylated interferon α-2b; NAs-CHB, chronic hepatitis B patients receiving nucleos(t)ide analogues, TN-CHB, Treatment-naïve chronic hepatitis B patients, participants without HBV infection, Non-chronic hepatitis B control; BMI, body mass index; PHQ-9, Patient Health Questionnaire-9; GAD-7, Generalized Anxiety Disorder Scale-7.

### 3.2 Prevalence of male sexual dysfunction

Analysis of erectile function showed a graded pattern across the study groups, with the poorest erectile function observed in the IFN-CHB group. The overall distribution of EHS grades differed significantly among the IFN-CHB, NAs-CHB, TN-CHB, and non-HBV groups (*χ*^2^ = 40.21, *p* < 0.001), particularly in the proportions of participants with EHS grades I, II, and IV. A similar pattern was observed for IIEF-5 scores, which differed significantly among the four groups (*H* = 50.97, *p* < 0.001; Figure 1a and Supplementary Table S3). Median IIEF-5 scores were lowest in the IFN-CHB group and highest in non-HBV controls. IIEF-5 scores were significantly lower in the IFN-CHB group than in all other groups, and lower in the NAs-CHB group than in Non-HBV controls. Using the validated IIEF-5 cutoff of ≤21, ED prevalence was highest in the IFN-CHB group at 70.9% (214/302), followed by the NAs-CHB group at 55.8% (82/147), the TN-CHB group at 42.9% (18/42), and Non-HBV controls at 37.3% (31/83). In contrast, no significant intergroup differences were observed in PEDT scores (*H* = 2.18, *p* = 0.535; Figure 1b).

**Figure 1:**
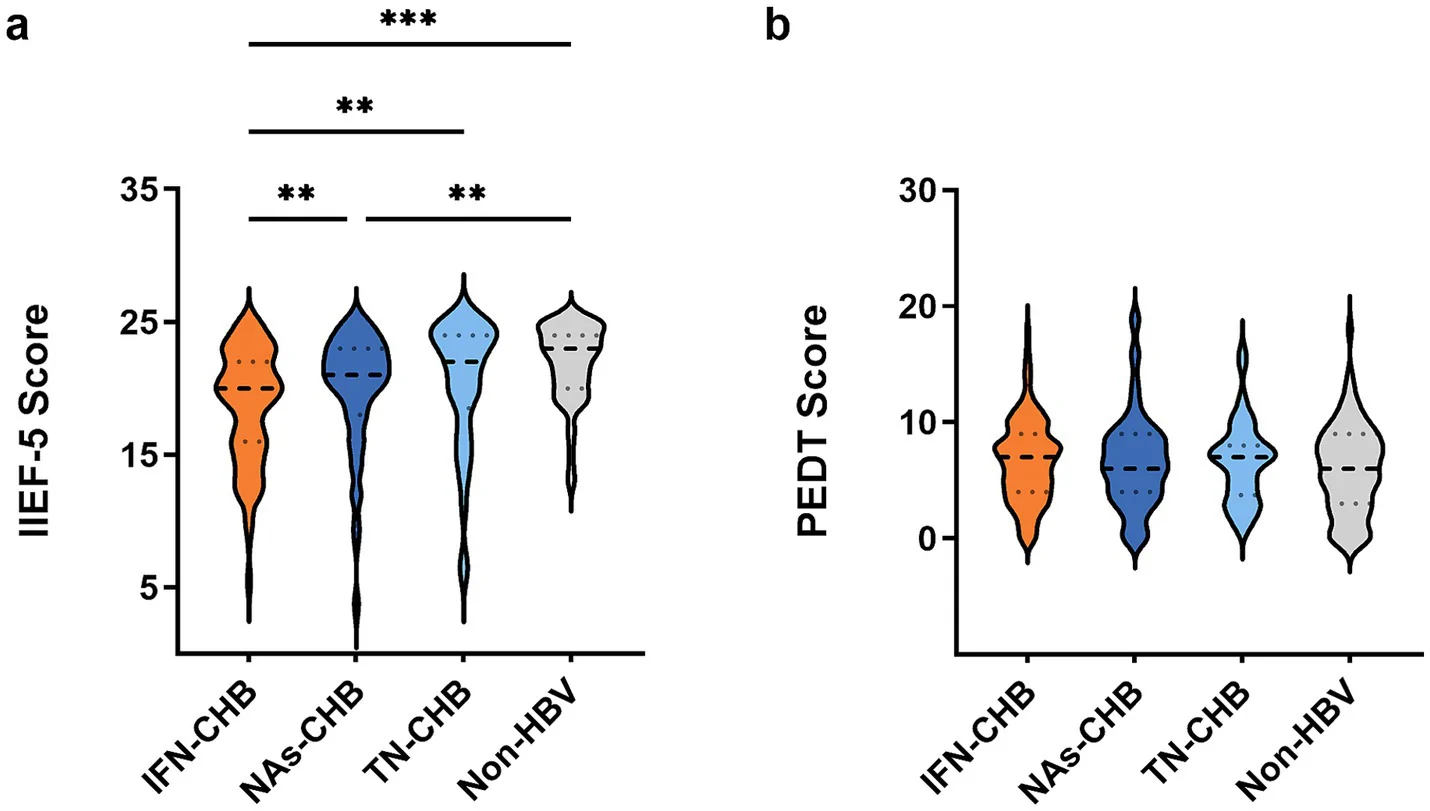
Sexual function scores in male participants across study groups. **(a)** Comparison of IIEF-5 scores among different subgroups of male hepatitis B patients and the control group. Significant reductions in IIEF-5 scores were observed in IFN-CHB group compared with the others; **(b)** Comparison of PEDT scores across the four groups, with no significant differences detected. IIEF-5, International Index of Erectile Function-5; PEDT, The Premature Ejaculation Diagnostic Tool, IFN-CHB, chronic hepatitis B patients receiving pegylated interferon α-2b; NAs-CHB, chronic hepatitis B patients receiving nucleos(t)ide analogues, TN-CHB, Treatment-naïve chronic hepatitis B patients, participants without HBV infection, Non-chronic hepatitis B control; *p < 0.05; **p < 0.01; ***p < 0.001.

### 3.3 Prevalence of female sexual dysfunction

Female sexual function, as measured by the FSFI, exhibited a pattern consistent with the male cohort, though with some domain-specific nuances. The median FSFI total score was significantly lower in the IFN-CHB group [23.30 (18.70, 27.05)] compared with Non-HBV controls [27.60 (23.28, 31.00) (adjusted *p* = 0.004)]. FSD prevalence differed significantly among the four groups (*χ*2 = 12.08, *p* = 0.007). Notably, the IFN-CHB group had a higher prevalence of FSD than Non-HBV controls (70.8% vs. 39.5%, adjusted *p* = 0.003). Domain-level analyses provided deeper insight into the nature of this dysfunction. While desire and satisfaction scores were comparable across groups, the IFN-CHB group scored significantly lower than the Non-HBV group in aspects of arousal, lubrication, orgasm, and pain (all *p* < 0.05, Figure 2 and Supplementary Table S3).

**Figure 2:**
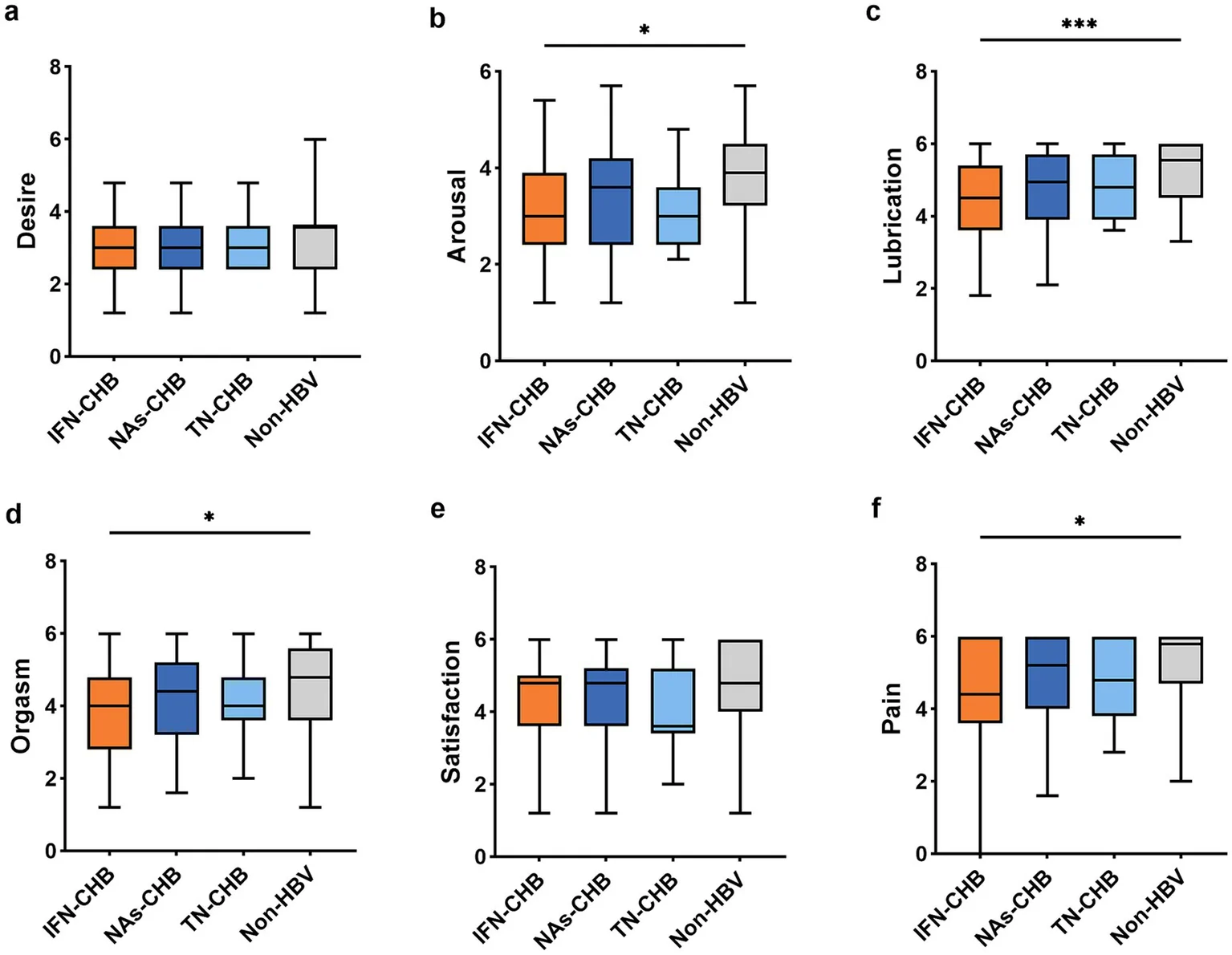
Distribution of FSFI domain scores in the research population. **(a,e)** No significant differences in sexual desire or satisfaction were observed among the four groups. **(b,c,d,f)** Statistically significant differences were observed between the IFN-CHB and Non-HBV groups in terms of sexual arousal, lubrication, orgasm and pain. IFN-CHB, chronic hepatitis B patients receiving pegylated interferon α-2b; NAs-CHB, chronic hepatitis B patients receiving nucleos(t)ide analogues, TN-CHB, Treatment-naïve chronic hepatitis B patients, participants without HBV infection, Non-chronic hepatitis B control; *p < 0.05; **p < 0.01; ***p < 0.00.

### 3.4 Potential factors associated with sexual function

Results of the univariable analyses are presented in Table 2. Hierarchical multiple linear regression analyses were performed separately in men and women to examine factors associated with sexual function scores. In men, older age (*β* = −0.125, *p* = 0.002), interferon-based treatment (*β* = −0.267, *p* < 0.001), and higher PHQ-9 score (*β* = −0.253, *p* < 0.001) were independently associated with lower IIEF-5 scores (Table 3). Of these, IFN-CHB status showed the strongest negative association, followed by PHQ-9 score. In women, older age (*β* = −0.172, *p* = 0.012), alcohol consumption (*β* = −0.183, *p* = 0.047), interferon-based treatment (*β* = −0.221, *p* = 0.018), and higher PHQ-9 score (*β* = −0.312, *p* = 0.006) were independently associated with lower FSFI scores (Table 4). Of these, PHQ-9 score showed the strongest negative association, followed by IFN-CHB status. In linear mixed-effects models with site as a random intercept, the factors identified in the primary analyses remained significantly associated with sexual function, except for IFN-CHB in women. The site-level ICC was <0.001 in both models, indicating minimal residual clustering by recruitment site. Detailed results are presented in Supplementary Table S5.

**Table 2:** Univariate analysis of sexual dysfunction in chronic hepatitis B patients.

Characteristics	Male		Female	
	*β* (95%CI)	*P* value	*β* (95%CI)	*P* value
Age, per year	−0.075 (−0.12, −0.03)	0.001	−0.099 (−0.20, 0.00)	0.052
Body mass index (BMI), kg/m^2^	0.061 (−0.06, 0.19)	0.340	−0.103 (−0.32, 0.12)	0.355
Education level (vs. High school)				
Secondary school	−1.775 (−2.85, −0.70)	0.001	0.013 (−2.62, 2.64)	0.992
University or above	1.372 (0.59, 2.15)	0.001	1.925 (0.16, 3.69)	0.033
Marital status (vs. Married)				
Unmarried Divorced	−0.807 (−2.17, 0.55) −0.168 (−2.91, 2.57)	0.244 0.904	0.662 (−3.26, 4.59) −0.271 (−4.20,3.65)	0.740 0.892
Smoking (Yes vs. No)	−0.151 (−1.10, 0.79)	0.754	−10.40 (−16.01, −4.78)	<0.001
Alcohol use (Yes vs. No)	−0.156 (−2.02,1.71)	0.870	−15.67 (−23.53, −7.81)	<0.001
Regular exercise (Yes vs. No)	0.226 (−0.59, 1.04)	0.587	0.546 (−1.35, 2.44)	0.570
Treatment group (vs. Non-HBV)				
IFN-CHB NAs-CHB TN-CHB	−3.259 (−4.29, −2.23) −1.962 (−3.10, −0.82) −1.452 (−3.02, 0.12)	<0.001 0.001 0.070	−3.755 (−5.93, −1.59) −2.050 (−4.41, 0.30) −2.490 (−5.54, 0.56)	0.001 0.087 0.108
Duration of HBV diagnosis, years	0.028(−0.01, 0.07)	0.151	−0.056(−0.15, 0.04)	0.233
Perceived infectivity impact (vs. Non-HBV)				
Negative impact No impact	−4.333(−5.54, −3.13) −2.271(−3.27, −1.27)	<0.001 <0.001	−5.017(−7.48, −2.55) −2.322(−4.38, −0.27)	<0.001 0.027
PHQ-9 score, per point	−0.181(−0.25, −0.11)	<0.001	−0.269(−0.42, −0.12)	0.001
GAD-7 score, per point	−0.123(−0.20, −0.04)	0.003	−0.171(−0.35, 0.01)	0.062

IFN-CHB, chronic hepatitis B patients receiving pegylated interferon α-2b; NAs-CHB, chronic hepatitis B patients receiving nucleos(t)ide analogues, TN-CHB, Treatment-naïve chronic hepatitis B patients, participants without HBV infection, Non-chronic hepatitis B control; BMI, body mass index; PHQ-9, Patient Health Questionnaire-9; GAD-7, Generalized Anxiety Disorder Scale-7.

**Table 3:** Multiple linear regression analysis of sexual function in male patients with hepatitis B.

Characteristics	Model 1		Model 2		Model 3	
	*β* (95% CI)	*P* value	*β* (95% CI)	*P* value	*β* (95% CI)	*P* value
Age, per year	−0.088 (−0.09,−0.01)	0.033	−0.099 (−0.10,−0.01)	0.016	−0.125 (−0.11,−0.02)	0.002
Education level (vs. High school)						
Secondary school	−0.143 (−2.79,−0.65)	0.002	−0.146(−2.81,−0.71)	0.001	−0.139 (−2.71,−0.64)	0.002
University or above	0.139 (0.43,2.01)	0.003	0.080 (−0.11,1.51)	0.090	0.067 (−0.22,1.39)	0.156
Treatment group (vs. Non-HBV)						
IFN-CHB			−0.294 (−3.62,−1.51)	<0.001	−0.267 (−3.38,−1.29)	<0.001
NAs-CHB			−0.105 (−2.22,0.12)	0.079	−0.096 (−2.11,0.19)	0.102
TN-CHB			−0.073 (−2.76,0.33)	0.123	−0.060 (−2.53,0.51)	0.193
PHQ-9 score, per point					−0.253 (−0.34,−0.11)	<0.001
GAD-7 score, per point					0.086 (−0.04,0.21)	0.190
R^2^	0.075		0.121		0.157	
ΔR^2^	0.075		0.046		0.035	

The maximum VIF in the final model was 2.868. *β* values are standardized regression coefficients. Abbreviations: IFN-CHB, chronic hepatitis B patients receiving pegylated interferon α-2b; NAs-CHB, chronic hepatitis B patients receiving nucleos(t)ide analogues, TN-CHB, Treatment-naïve chronic hepatitis B patients, participants without HBV infection, non-chronic hepatitis B control; PHQ-9, patient health questionnaire-9; GAD-7, Generalized Anxiety Disorder Scale-7.

**Table 4:** Multiple linear regression analysis of sexual function in female patients with hepatitis B.

Characteristics	Model 1		Model 2		Model 3	
	*β* (95% CI)	*P* value	*β* (95% CI)	*P* value	*β* (95% CI)	*P* value
Age, per year	−0.126 (−0.19,−0.01)	0.069	−0.147 (−0.21,−0.10)	0.033	−0.172 (−0.22,−0.03)	0.012
Education level (vs. High school)						
Secondary school	−0.015(−2.82,2.31)	0.843	−0.002 (−2.49,2.58)	0.975	0.038 (−1.83,3.15)	0.604
University or above	0.106 (0.50,2.96)	0.163	0.670 (−0.98,2.54)	0.385	0.090 (−0.67,2.77)	0.229
Smoking (Yes vs. No)	−0.129 (−13.16,2.40)	0.174	−0.141 (−13.58,1.80)	0.132	−0.094 (−11.55,3.73)	0.314
Alcohol use (Yes vs. No)	−0.168 (−20.85,1.06)	0.076	−0.144 (−19.27,2.36)	0.125	−0.183 (−21.39,−0.14)	0.047
Treatment group (vs. Non-HBV)						
IFN-CHB			−0.282 (−5.45,−1.14)	0.003	−0.221 (−4.71,−0.45)	0.018
NAs-CHB			−0.124 (−3.95,0.74)	0.178	−0.072 (−3.25,1.39)	0.431
TN-CHB			−0.121 (−5.33,0.72)	0.134	−0.103 (−4.91,1.00)	0.193
PHQ-9 score, per point					−0.312 (−0.60,−0.10)	0.006
GAD-7 score, per point					0.088(−0.17,0.40)	0.428
R^2^	0.112		0.155		0.211	
ΔR^2^	0.112		0.042		0.056	

The maximum VIF in the final female model was 3.042. β values are standardized regression coefficients. IFN-CHB, chronic hepatitis B patients receiving pegylated interferon α-2b; NAs-CHB, chronic hepatitis B patients receiving nucleos(t)ide analogues, TN-CHB, Treatment-naïve chronic hepatitis B patients, participants without HBV infection, Non-chronic hepatitis B control; PHQ-9, Patient Health Questionnaire-9; GAD-7, Generalized Anxiety Disorder Scale-7.

### 3.5 Association between perceived negative impact of HBV infectivity and sexual function

In this study, the perceived negative impact of HBV infectivity referred to patients’ self-reported perception that the infectious nature of HBV negatively affected their sexual life. Because this HBV-specific item was not applicable to the Non-HBV group, it was not considered a candidate variable for the primary whole-sample multivariable models and was analyzed separately (Figure 3). Among patients with CHB, 21.6% of men (106/491) and 24.7% of women (41/166) reported a perceived negative impact of HBV infectivity on sexual life. Men reporting this perception had significantly lower IIEF-5 scores than those who did not (*p* < 0.001), and women reporting this perception also had lower FSFI total scores (*p* = 0.020).

**Figure 3:**
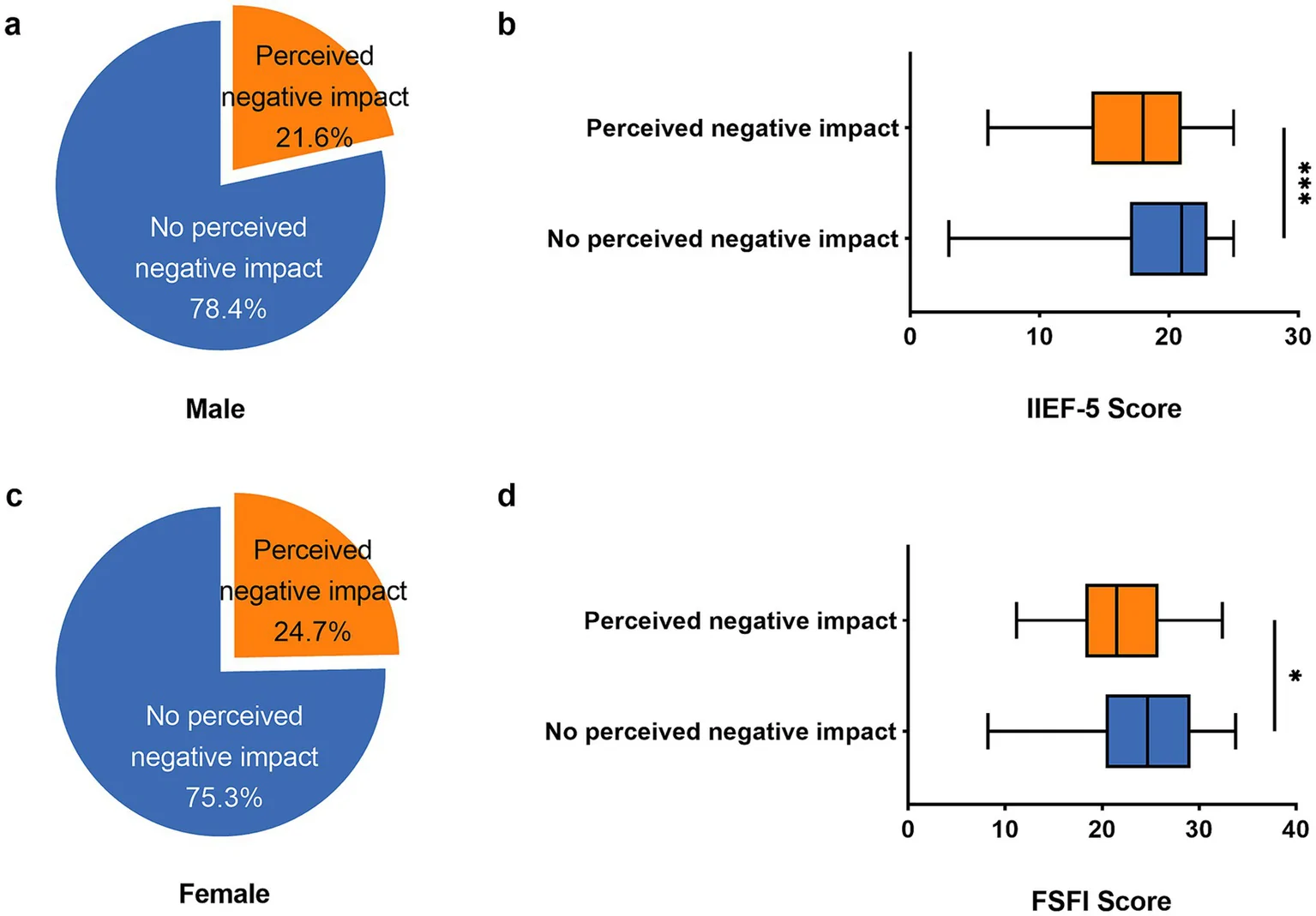
Association between perceived negative impact of HBV infectivity and sexual function. **(a)** Proportion of male participants reporting a perceived negative impact of HBV infectivity; **(b)** Comparison of IIEF-5 scores between males with and without perceived negative impact; **(c)** Proportion of female participants reporting a perceived negative impact of HBV infectivity; **(d)** Comparison of FSFI scores between females with and without perceived negative impact. IIEF-5, International Index of Erectile Function-5; FSFI, Female Sexual Function Index; *p < 0.05; **p < 0.01; ***p < 0.001.

### 3.6 Prevalence and factors associated with sexual inactivity

Among men, sexual inactivity differed across the four groups and was most prevalent in IFN-CHB group (12.7%), followed by Non-HBV group (3.5%), NAs-CHB group (2.6%), and TN-CHB group (0%) (*p* < 0.001; Supplementary Table S2). In multivariable analysis, unmarried status (OR 11.58, *p* < 0.001) and a higher PHQ-9 score (OR 1.11, *p* = 0.021) were associated with sexual inactivity, whereas IFN-CHB status did not reach statistical significance (OR 2.93, *p* = 0.051; Supplementary Table S4). Among women, the prevalence of sexual inactivity was 35.0% in IFN-CHB group, 27.3% in NAs-CHB group, 19.2% in TN-CHB group, and 19.1% in Non-HBV group (*p* = 0.113; Supplementary Table S2). Older age (OR 1.05, *p* = 0.005) and unmarried status (OR 7.76, *p* < 0.001) were associated with sexual inactivity, whereas IFN-CHB status was not significantly associated with this outcome (OR 2.04, *p* = 0.114; Supplementary Table S4).

## 4 Discussion

This study provides a comprehensive characterization of the burden of SD among patients with CHB. We observed a high prevalence of SD among sexually active patients with CHB, ranging from 42.9 to 70.9% for ED in men and from 58.9 to 70.8% for FSD in women. A large Chinese study using the ASEX reported corresponding estimates of 20.0 and 57.7% in men and women with CHB (10). However, substantial heterogeneity in prevalence estimates was reported in a recent meta-analysis (I^2^ > 94%), underscoring the limited comparability of findings across studies with different participant characteristics, assessment instruments, and diagnostic thresholds (4). In multivariable analyses, older age, IFN-CHB status, and depressive symptoms (higher PHQ-9 scores) were associated with poorer sexual function. These findings support a biopsychosocial perspective on sexual dysfunction in CHB. Sex-stratified results provided further insight into the patterns of SD in CHB. In men, the excess burden was primarily reflected in ED. In women, group differences were observed mainly in arousal, lubrication, orgasm, and pain domains, consistent with findings in other chronic conditions (22). Interestingly, the factor most associated with poorer sexual function in our study was depressive symptoms in women, and IFN-CHB status in men. These descriptive patterns may partly reflect the multidimensional effects of depressive symptoms on female sexual function, the potential systemic effects of interferon treatment on erectile function, and differences between the constructs measured by the FSFI and IIEF-5.

Age was associated with poorer sexual function in both men and women, which is consistent with the established influence of aging on sexual health (23). However, age alone did not fully explain the observed differences across treatment groups. After adjustment for age and other covariates, IFN-CHB status and higher PHQ-9 scores remained associated with lower sexual function scores, and similar patterns were observed in age-stratified analyses. These findings suggest that age should be considered an important background factor, but not the sole explanation for sexual dysfunction in patients with CHB.

The association between pegylated interferon α-2b therapy and poorer sexual function was one of the most notable findings of this study. Common adverse effects of Peg-IFN α-2b include fatigue, myalgias, fever and mood-related symptoms (24), which could plausibly interfere with sexual interest and functioning. In addition, studies in hepatitis C populations have reported that interferon α may affect the hypothalamic pituitary gonadal axis and sex hormone profiles, including reduced testosterone levels in some male patients (25, 26). This endocrine pathway may also be present in our IFN-CHB group and partly explain the poorer erectile function observed in these patients. In contrast, NAs were not independently associated with sexual function in the final multivariable model, despite a high prevalence of SD in the NAs-CHB group, aligns with recent studies suggesting that new-generation NAs (e.g., entecavir, tenofovir) have a neutral impact on sexual function (11, 12). This implies that the dysfunction observed in the NAs-CHB group likely reflects the systemic burden of chronic disease rather than a drug-specific pharmacologic toxicity.

Depressive symptoms also emerged as an important correlate of poorer sexual function, as reflected by the independent association between PHQ-9 scores and lower IIEF-5 and FSFI scores. This finding is consistent with previous evidence linking depressive symptoms to impaired sexual function (6, 27) and suggests that sexual health problems in CHB should be considered within a broader psychosocial context. However, given that interferon therapy may induce or exacerbate depressive symptoms, it remains unclear whether the association between depressive symptoms and sexual dysfunction is independent of the treatment or is in part attributable to an interferon-related pathway. HBV infection carries a distinctive stigma related to its infectivity. Patients may fear transmitting the virus to intimate partners, and such concerns may contribute to reduced sexual confidence, relationship-related anxiety, and avoidance of sexual activity (28, 29). This interpretation is further supported by our stratified analysis, which showed that patients perceived negative impact of HBV infectivity on sexual life had lower sexual function scores. Thus, beyond treatment status and depressive symptoms, perceived transmission risk may represent a specific psychosocial barrier to sexual well-being. Notably, although GAD-7 scores were associated with sexual function in univariable analysis, this association did not persist after multivariable adjustment, suggesting that anxiety-related distress may overlap with depressive symptoms in this context.

Notably, a considerable proportion of participants reported sexual inactivity, with the highest prevalence observed in the IFN-CHB group. This trend was consistent with primary analysis. Among men, the proportion in the IFN-CHB group was 12.7%, slightly higher than age-comparable estimates from the US general population (30). Among women, sexual inactivity was more common overall, prevalence reached 35.0% in the IFN-CHB group and 27.3% in the NAs-CHB group, exceeding US estimates but closer to Japanese estimates (31, 32). In multivariable analyses, the adjusted associations between IFN-CHB and sexual inactivity remained positive in both sexes but did not reach statistical significance. The limited number of events, particularly within sex-specific treatment groups, may have reduced the precision of these estimates. Unmarried status and depressive symptoms were associated with sexual inactivity in men, and older age and unmarried status in women. These factors only partly overlapped with those associated with sexual function scores, likely reflecting the broader determinants of sexual inactivity. Sexual function scores assess functioning among participants with recent sexual activity. Yet sexual inactivity may particularly reflect partner availability and relationship circumstances, as well as psychological distress or impaired sexual function (33, 34). Therefore, sexual inactivity should be regarded as a complementary sexual health outcome rather than a proxy for sexual dysfunction.

This study has several limitations. First, its cross-sectional design precludes causal inference. Nonrandom treatment selection may also have introduced confounding because patients receiving Peg-IFN α-2b, NAs, or no antiviral therapy may differ in clinical characteristics, disease activity, treatment expectations, and pretreatment psychological or sexual function. The modest R^2^ values further suggest that relevant determinants were not captured by the models. Second, the absence of objective biomarkers, specifically serum testosterone levels, luteinizing hormone (LH), and follicle-stimulating hormone (FSH), precluding assessment of possible HPG axis dysregulation. Although patients with documented cirrhosis or major comorbidities were excluded, systematic fibrosis assessments and liver biochemistry data were unavailable. Therefore, residual differences in liver disease severity may have confounded the associations between treatment status and sexual function. Third, although the Non-HBV group provided a reference for comparison, they were not individually matched to CHB patients. Thus, despite statistical adjustment, demographic differences, particularly in education level, may not have been fully accounted for. Fourth, the sample size calculation targeted the precision of the overall prevalence estimate among patients with CHB rather than sex-by-treatment-group comparisons. Small strata, particularly among women, may therefore have reduced estimate precision and the power to detect modest differences. Finally, self-reported sexual-function and psychological measures, which may be affected by recall bias, social desirability bias, and cultural sensitivity surrounding sexual health. The perceived negative impact of HBV infectivity on sexual life was assessed using a single yes/no item, which may not fully capture the complexity of infectivity-related concerns. Participants who reported no sexual activity in the previous 3 months were excluded, which may have underestimated the true prevalence of sexual dysfunction.

Despite these limitations, our findings highlight the potential value of addressing sexual health as part of comprehensive CHB care. Sexual health assessment may help identify unmet care needs that are not captured by routine virological or biochemical monitoring. For patients scheduled to start interferon-based therapy, identifying individuals with pre-existing depressive symptoms (high PHQ-9 scores) or heightened concerns about HBV transmission allows for proactive counseling. Discussing the potential for interferon-associated fatigue and SD before treatment may help patients develop realistic expectations, reduce the risk of treatment discontinuation due to adverse effects, and facilitate timely referrals to mental health professionals. However, further evidence from adequately powered prospective interventional studies is needed to establish the clinical benefits of these approaches before widespread implementation.

## Data Availability

The raw data supporting the conclusions of this article will be made available by the authors, without undue reservation.
