# Supplementary material for: Sexual dysfunction, depressive symptoms, and perceived impact of HBV infectivity among noncirrhotic patients with chronic hepatitis B: a cross-sectional study
Source: Front Public Health. 2026 Sep 29;14:1906589. doi: 10.3389/fpubh.2026.1906589 (PMC13658144; doi:10.3389/fpubh.2026.1906589)
Supplement: Supplementary file 1 [file Supplementary_file_1.docx]

**Table S1. Participants initially recruited and included in the primary analytic sample by site**

| Site | Initially recruited, n | Included, n |
| --- | --- | --- |
| West China Hospital of Sichuan University | 496 | 415 |
| Public Health Clinical Center of Chengdu | 159 | 133 |
| Yangtze River Shipping General Hospital | 73 | 52 |
| Sichuan Provincial People’s Hospital | 64 | 45 |
| Tianjin Second People’s Hospital | 46 | 34 |
| Southwest Hospital, Army Medical University | 45 | 35 |
| Deyang People’s Hospital | 43 | 34 |
| The Second Affiliated Hospital of Xi’an Jiaotong University | 36 | 30 |
| Total | 962 | 778 |

Note: The 184 individuals not included in the primary analysis comprised 134 reporting no sexual activity in the previous three months, 42 with duplicate or invalid questionnaires, and 8 with major comorbidities as defined in the Methods section.

**Table S2 Sensitivity analyses accounting for sexual inactivity**

| Sex | Outcome | IFN-CHB | NAs-CHB | TN-CHB | Non-HBV | *P* value |
| --- | --- | --- | --- | --- | --- | --- |
| Men | Sexual inactivity | 44/346(12.7%) | 4/151(2.6%) | 0/42(0.0%) | 3/86(3.5%) | <0.001 |
|  | ED/sexual inactivity | 258/346(74.6%) | 85/151(56.3%) | 18/42(42.9%) | 34/86(39.5%) | <0.001 |
| Women | Sexual inactivity | 48/137(35.0%) | 21/77(27.3%) | 5/26(19.2%) | 9/47(19.1%) | 0.113 |
|  | FSD/sexual inactivity | 111/137(81.0%) | 54/77(70.1%) | 20/26(76.9%) | 24/47(51.1%) | 0.001 |

Note: Sexual inactivity was defined as self-reported no sexual activity in the previous three months. Abbreviations: IFN-CHB, chronic hepatitis B patients receiving pegylated interferon α-2b; NAs-CHB, chronic hepatitis B patients receiving nucleos(t)ide analogues, TN-CHB, Treatment-naïve chronic hepatitis B patients, Non-HBV, participants without HBV infection; ED, erectile dysfunction; FSD, female sexual dysfunction.

**Table S3 Overall and pairwise comparisons of sexual function scores across study groups**

| Sexual function | Kruskal-Wallis test, *P* value | Bonferroni-adjusted *P* values for pairwise comparisons | | | | | |
| --- | --- | --- | --- | --- | --- | --- | --- |
|  |  | IFN-CHB vs NAs-CHB | IFN-CHB vs TN-CHB | IFN-CHB vs Non-HBV | NAs-CHB vs TN-CHB | NAs-CHB vs Non-HBV | TN-CHB vs Non-HBV |
| IIEF-5 score | <0.001 | 0.003 | 0.004 | <0.001 | 1.000 | 0.004 | 1.000 |
| FSFI total score | 0.006 | 0.401 | 1.000 | 0.004 | 1.000 | 0.573 | 0.395 |
| Desire | 0.228 |  |  |  |  |  |  |
| Arousal | 0.032 | 1.000 | 1.000 | 0.023 | 1.000 | 0.453 | 0.278 |
| Lubrication | <0.001 | 0.182 | 0.636 | <0.001 | 1.000 | 0.150 | 0.594 |
| Orgasm | 0.053 |  |  |  |  |  |  |
| Satisfaction | 0.045 | 1.000 | 1.000 | 0.055 | 1.000 | 0.260 | 0.146 |
| Pain | 0.031 | 0.484 | 1.000 | 0.026 | 1.000 | 1.000 | 1.000 |

Note: Post hoc pairwise comparisons were performed only when the overall test was statistically significant, with P values adjusted using Bonferroni correction. Abbreviations: IIEF-5, International Index of Erectile Function-5; FSFI, Female Sexual Function Index; IFN-CHB, chronic hepatitis B patients receiving pegylated interferon α-2b; NAs-CHB, chronic hepatitis B patients receiving nucleos(t)ide analogues, TN-CHB, Treatment-naïve chronic hepatitis B patients, Non-HBV, participants without HBV infection.

**TableS4 Multivariable logistic regression analyses of sexual inactivity in men and women**

| Characteristics | Male | | Female | |
| --- | --- | --- | --- | --- |
|  | Adjusted OR (95% CI) | *P* value | Adjusted OR (95% CI) | *P* value |
| Age, per year | 1.03(0.99,1.08) | 0.137 | 1.05(1.01,1.08) | 0.005 |
| Education Level (vs. High school)  Secondary school  University or above | 1.59(0.62,3.95)  1.20(0.58,2.57) | 0.327  0.628 | 0.78(0.31,1.95)  0.93(0.49,1.77) | 0.596  0.819 |
| Marital Status (vs. Married)  Non-married | 11.58(5.29,26.14) | <0.001 | 7.76(3.60,16.73) | <0.001 |
| Treatment Group (vs. Non‑HBV)  IFN-CHB  NAs-CHB  TN-CHB | 2.93(0.99,11.54)  0.62(0.13,3.06)  0.16(0.00,1.08) | 0.051  0.536  0.160 | 2.04(0.84,4.95)  1.56(0.59,4.14)  0.95(0.25,3.61) | 0.114  0.367  0.936 |
| PHQ‑9 score, per point | 1.11(1.02,1.21) | 0.021 | 0.99(0.91,1.08) | 0.859 |
| GAD‑7 score, per point | 0.95(0.85,1.06) | 0.388 | 1.07(0.96,1.18) | 0.211 |

Note: Firth’s penalized likelihood logistic regression was used for men because no events occurred in the TN-CHB group. Marital status was dichotomized as married versus non-married because of sparse observations in some original categories. The non-married category included participants who were never married, divorced, or widowed. Abbreviations: IFN-CHB, chronic hepatitis B patients receiving pegylated interferon α-2b; NAs-CHB, chronic hepatitis B patients receiving nucleos(t)ide analogues, TN-CHB, Treatment-naïve chronic hepatitis B patients, Non-HBV, participants without HBV infection; PHQ-9, Patient Health Questionnaire-9; GAD-7, Generalized Anxiety Disorder Scale-7.

**TableS5 Linear mixed-effects analyses of sexual function accounting for clustering within recruitment sites**

| Characteristics | Male | | Female | |
| --- | --- | --- | --- | --- |
|  | β (95%CI) | *P* value | β (95%CI) | *P* value |
| Age, per year | -0.125(-0.11, -0.02) | 0.002 | -0.172(-0.23, -0.03) | 0.013 |
| Education Level **(vs. High school)**  **Secondary school**  University or above | -0.139(-2.71, -0.64)  0.067(-0.23, 1.39) | 0.002  0.157 | 0.038(-1.84, 3.16)  0.091(-0.68, 2.79) | 0.605  0.231 |
| Smoking (Yes vs. No) |  |  | -0.094(-11.59, 3.78) | 0.317 |
| Alcohol use (Yes vs. No) |  |  | -0.183(-21.71, 0.17) | 0.054 |
| **Treatment Group (vs. Non‑HBV)**  IFN‑CHB  NAs‑CHB  TN‑CHB | -0.267(-3.86, -0.81)  -0.096(-2.11, 0.19)  -0.060(-2.53, 0.51) | 0.005  0.102  0.193 | -0.221(-6.94, 1.78)  -0.071(-3.25, 1.40)  -0.102(-4.90,1.02) | 0.182  0.432  0.197 |
| PHQ‑9 score, per point | -0.253(-0.34, -0.11) | <0.001 | -0.312(-0.61, -0.10) | 0.006 |
| GAD‑7 score, per point | 0.086(-0.04, 0.21) | 0.190 | 0.088(-0.18, 0.41) | 0.441 |

Note: β values are standardized regression coefficients. The estimated site-level ICC was <0.001 in both models. Abbreviations: IFN-CHB, chronic hepatitis B patients receiving pegylated interferon α-2b; NAs-CHB, chronic hepatitis B patients receiving nucleos(t)ide analogues, TN-CHB, Treatment-naïve chronic hepatitis B patients, Non-HBV, participants without HBV infection; PHQ-9, Patient Health Questionnaire-9; GAD-7, Generalized Anxiety Disorder Scale-7; ICC, intraclass correlation coefficient.
